# Duck plague virus Glycoprotein J is functional but slightly impaired in viral replication and cell-to-cell spread

**DOI:** 10.1038/s41598-018-22447-x

**Published:** 2018-03-06

**Authors:** Yu You, Tian Liu, Mingshu Wang, Anchun Cheng, Renyong Jia, Qiao Yang, Ying Wu, Dekang Zhu, Shun Chen, Mafeng Liu, XinXin Zhao, Shaqiu Zhang, Yunya Liu, Yanling Yu, Ling Zhang

**Affiliations:** 1 Institute of Preventive Veterinary Medicine, Sichuan Agricultural University, Wenjiang, Chengdu, Sichuan 611130 P.R. China; 2 Key Laboratory of Animal Disease and Human Health of Sichuan Province, Sichuan Agricultural University, Wenjiang, Chengdu, Sichuan 611130 P.R. China; 3 Avian Disease Research Center, College of Veterinary Medicine, Sichuan Agricultural University, Wenjiang, Chengdu, Sichuan 611130 P.R. China

## Abstract

To analyse the function of the duck plague virus (DPV) glycoprotein J homologue (gJ), two different mutated viruses, a gJ deleted mutant ΔgJ and a gJR rescue mutant gJR with US5 restored were generated. All recombinant viruses were constructed by using two-step of RED recombination system implemented on the duck plague virus Chinese virulent strain (DPV CHv) genome cloned into a bacterial artificial chromosome. DPV-mutants were characterized on non-complementing DEF cells compared with parental virus. Viral replication kinetics of intracellular and extracellular viruses revealed that the ΔgJ virus produce a 10-fold reduction of viral titers than the gJR and parental virus, which especially the production of extracellular infectivity was affected. In addition, the ΔgJ virus produced viral plaques on DEF cells that was on average approximately 11% smaller than those produced by the gJR and parental viruses. Electron microscopy confirmed that although DPV CHv without gJ could efficiently carry out viral replication, virion assembly and envelopment within infected cells, the ΔgJ virus produced and accumulated high levels of anuclear particles in the nuclear and cytoplasm. These results show that the gJ slightly impaired in viral replication, virion assembly and cell-to-cell spread, and is not essential in virion envelopment.

## Introduction

Duck plague(DP), also called duck virus enteritis (DVE), is one of the major acute, fatal and contagious diseases of duck, geese, and swans, characterized by vascular damage, tissue hemorrhages, digestive mucosal eruptions, and lesions of lymphoid organs. Due to high mortality, morbidity as well as decreased egg production and hatchability, DP caused significant economic losses around the world^1–4^. DPV, which belong to the genus Mardivirus, subfamily Alpha-herpesvirinae, and family Herpesviridae, is the pathogen of DP disease^3,4^. The entire DNA sequence of the DPV has been determined few years ago^5^. However, not too many genes and gene products have been characterized in term of relevance or functional cooperation in viral lifecycle^6–8^.

Herpesvirus glycoproteins paly important roles in the different stages of viral lifecycle, such as enter the target cells, direct cell-to-cell spread, and the egress of virions from infected cells^9,10^, and 12 glycoproteins have been identified and designated gB, gC, gD, gE, gG, gH, gI, gJ, gK, gL, gM and gN in herpes simplex virus type 1 (HSV-1)^11^. DPV glycoproteins are named in accordance with the nomenclature used for the HSV-1 glycoproteins. However, previously studies about DPV focused on the epidemiology and prevention, instead of the studying of molecular biology and the function of glycoproteins^5^. To date only a few gens such as gC and gE from DPV have been addressed by studying mutant viruses^12,13^. gC involved in alphaherpesvirus adsorption were present in the DPV genome and relatively conserved, but the function of DPV gC also makes a difference with other alphaherpesvirus, which is independent of interaction with heparin sulfate of cell surface^12,14^. Therefore, although alphaherpesvirus share many common strategies for their replication cycle^15^, they also keep some individually patterns to invade and infect target cells.

The US5 gene of alpha-herpesvirus encodes glycoprotein J (gJ), which shares lowly nucleotide and amino acid similarity among alpha-herpesvirus sub-family. Exploring the functions of gJ is far behind other glycoproteins. The only previously reported function of alpha-herpesvirus gJ was its ability to inhibit apoptosis and viral egress, and the mechanisms of these functions have not been fully revealed^16–20^. DPV glycoprotein J (gJ) is encoded by US5 in the viral genome which is positional homologue in the HSV-1 genome. Comparisons of nucleotide and deduced amino acid sequences uncovered that gJ is lowly conserved throughout the alpha-herpesvirus subfamily, but highly conserved among the different strain of DPV.

In the present study, to clarify roles of gJ in DPV lifecycle, we describe the construction of a DPV mutant virus, based on the infectious BAC clone by using two-step of RED recombination for generation of US5 deletion and revertant mutant in *E.coli*. Viral particles lacking gJ in the envelope were produced in non-complementing DEF cells, which demonstrated that US5 gene of DPV is nonessential for virus replication. We determined kinetics of virus growth, relative plague morphology, and conducted ultrastructural visualization of gJ-deficient mutant and parental virions in the same DPV genetic background to gain an understanding of the glycoprotein J contribution in infectious virion replication cycle. The results firstly show that gJ is nonessential for DPV virion replication and slightly impaired in viral replication, virion assembly and cell-to-cell spread.

## Results

### Construction and molecular analysis of recombinant virus

**Figure 1:**
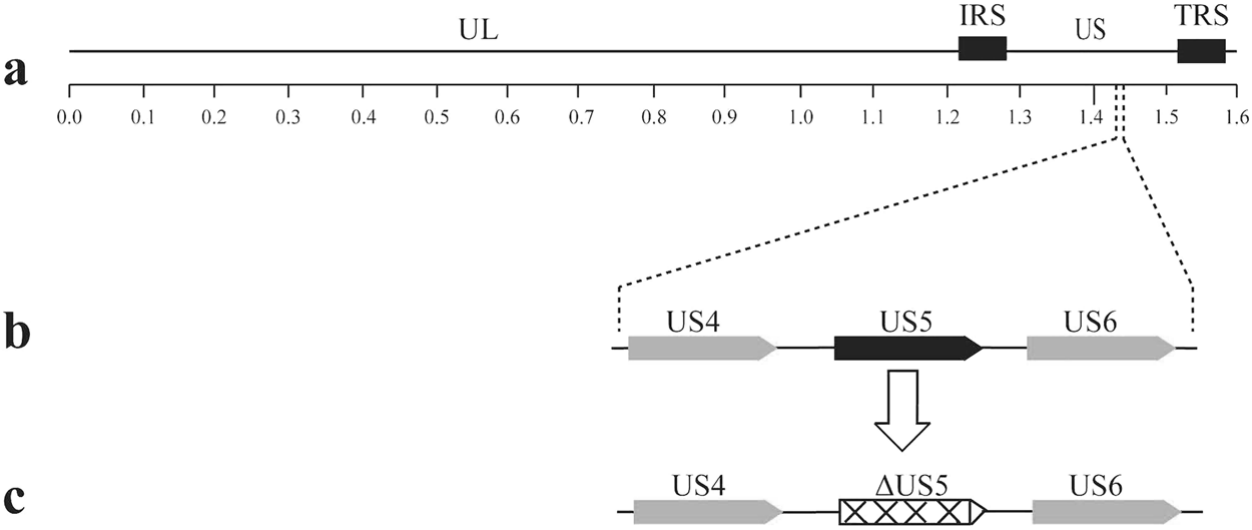
Genomic map of mutated genes. (**a**) Represents the prototypic arrangement of the DPV genome with the unique long (UL) and unique short (US) regions flanked by the terminal repeat (TR) and internal repeat (IR) regions. (**b**) Shows expanded genomic regions of the US4, US5, and US6 open reading frames. (**c**) Shows the deletion of the whole US5 open reading frames.

The DPV genome has been cloned into a BAC by our laboratory, which enables the rapid and efficient genetic manipulation of the DPV genome in *E. Coli DH10B*. To investigate of the relative role of DPV gJ, the gJ deletion mutant and its revertant was generated using the two-step Red recombination mutagenesis system implemented on the pBeloBAC11 bacterial artificial chromosome carrying the DPV genome, as described in Materials and Methods. The DPV CHv-BAC-ΔgJ virus is the deletion of the entire gJ open reading frame (ORF) (Fig. 1). Meanwhile, to eliminate the small possibility that this deletion causes indirect epigenetic effects on foreign DNA insertion, we constructed the DPV CHv-BAC-gJR virus which is the revertant of the entire gJ ORF.

**Figure 2:**
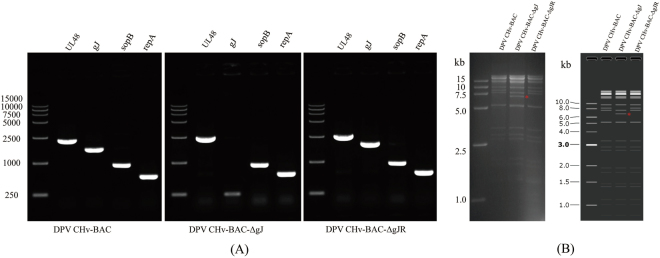
Identification of the recombinant viruses. (**A**) PCR analysis of the gJ deletion mutant. The BAC DNAs of DPV CHv-BAC, DPV CHv-BAC-ΔgJ and DPV CHv-BAC-ΔgJR were extracted and amplified by PCR using the indicated primers. The positions of DNA molecular size markers are shown on the lift side. (**B**) Restriction fragement length polymorphism analysis of recombinant virus. Indicated the orientation and real Gel analysis of DPV CHv-BAC, DPV CHv-BAC-ΔgJ and DPV CHv-BAC-gJR digested by *BamHI*, respectively. The asterisk was made to show the different band, and the image on the right was obtained by software simulation.

The engineered mutations were confirmed via diagnostic PCR, restriction fragment length polymorphism (RFLP) analysis and DNA sequencing (data not shown). Specifically, the BAC DNAs of DPV CHv-BAC-ΔgJ and DPV CHv-BAC-gJR were extracted, and then were identified by primer UL48 (2555 bp), gJ (1820 bp), sopB (966 bp) and repA (681 bp), among which UL48 is a DPV conversed gene to identify the existence of DPV genome and sopB as well as repA are two important genes to identify BAC-Mini-F sequence (Fig. 2A). As a result, we found repA, sopB and UL48 can be detected in all recombinant viruses, which indicated the presence of BAC component and DPV genome in recombinant viruses. What’s more, the whole fragment of gJ gene can be detected in the parental virus of DPV CHv-BAC and the revantant virus of DPV CHv-BAC-gJR, but cannot be found in the DPV CHv-BAC-ΔgJ, which showed that the construction of gJ-deleted DPV virus was successful.

Furthermore, the BAC DNA of recombinant virus extracted from the PCR identified clones was digested with *BamHI* for RFLP analysis. As a result, the restriction patterns of *BamHI* disgestion products of DPV CHv-BAC, DPV CHv-BAC-ΔgJ and DPV CHv-BAC-gJR were as same as we prediction, respectively (Fig. 2B).

### Rescue and confirm of the recombinant virus

**Figure 3:**
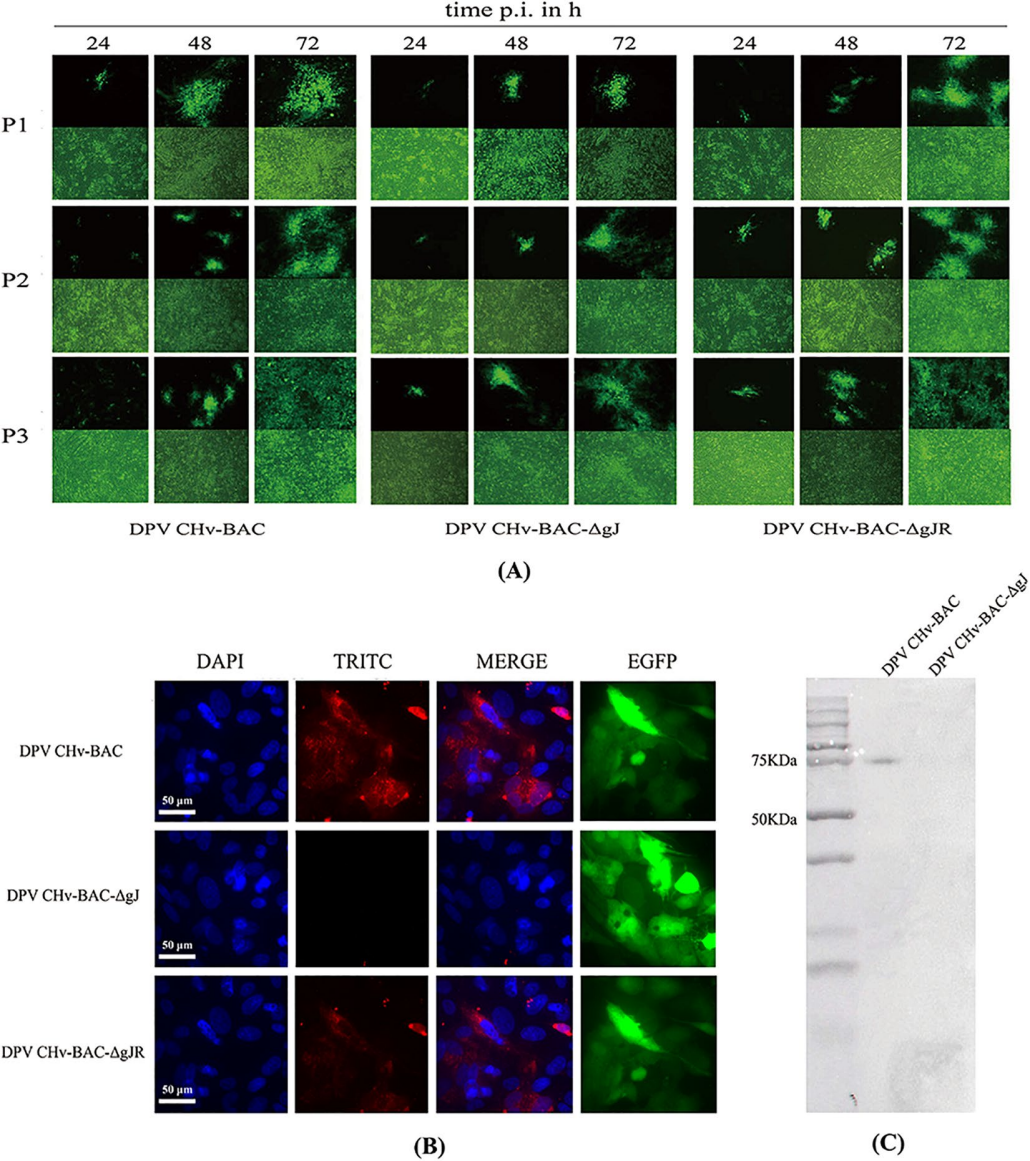
Rescue mutant viruses and Identification of gJ expression. (**A**) Purification and enrichment of mutant viruses. Purification and enrichment of mutant viruses were obtained by the three times passage after transfection. (**B**) Immunofluorescence detection of gJ expression. DEF cells were infected at 1000 TCID_50_, and gJ expression was detected by indirect immunofluorescence at 36 hpi. Rabbit anti-gJ were used as primary antibody, and goat anti-rabbit IgG TRITC were used as secondary antibody. (**C**) Anti-gJ monoclonal antibody (MAb) was used to detect gJ via western immunoblot analysis. DPV CHv-BAC-ΔgJ infected DEF cells were detected as parental virus.

To generate virus stocks from the mutant BAC genomic constructs, individual BAC DNAs were transfected into DEF cells. Passage of these viruses up to three times in DEF cells eliminated the effects of transient transfection of plasmid containing EGFP and did not led to any phenotypic revertants, which also purified and enriched the recombinant viruses (Fig. 3A).

The mutant viruses of DPV CHv-BAC-ΔgJ and DPV CHv-BAC-gJR were identified indirect immunouorescence assay (IFA) and western blotting. The expression of gJ protein in parental virus of DPV CHv-BAC and derived recombinant DPV CHv-BAC-ΔgJ and DPV CHv-BAC-gJR infected host cells were recongnized by polyclonal rabbit anti-gJ in IFA and WB (Fig. 3B,C).

### One-step viral growth kinetics

**Figure 4:**
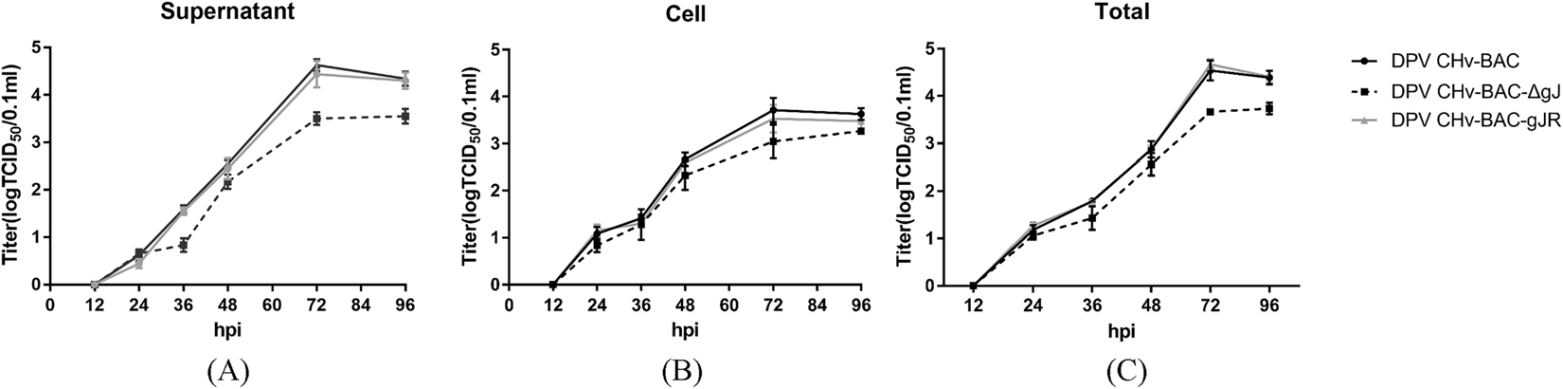
Replication kinetics of parental and mutant viruses. Confluent DEF cells monolayers were infected with each virus shown at an MOI of 0.01. Viral titer of infected supernatant, cells and mixture of cells cultures were determined at the indicated time points by measuring TCID_50_ on DEF cells. All titrations were carried out in three independent experiment. The titers obtained were averaged, and the standard error of the mean was calculated each time point.

To examine the effect of the gJ-deleted engineered mutation on virus replication, DEF cells were infected in an MOI of 0.01 with either the parental or each mutant viruses and virus growth kinetics within infected cells and their supernatants were performed as described in Materials and Methods. Compared to the parental virus, gJ-deleted mutant virus and its revantant virus accumulated infectious virions within infected cells at similar rates. As the result (Fig. 4), growth properties of the repaired virus DPV CHv-BAC-gJR resembled those of DPV CHv-BAC, whereas DPV CHv-BAC-ΔgJ presented with a marked growth defect. Within these experiments, the replication of viruses kept quiescence at the first 12 h after infection, then significant increases were observed during the whole observation time in supernatants while it stopped increasing at 72 hpi and slightly dropped after that in cells except DPV CHv-BAC-ΔgJ mutant virus. It was worthy to mention that viral titers in cells were higher than titers in medium at 24 hpi. However, on the contrary, at 48 hip, titers of mature viral particles in supernatant was more than virus in cells. Moreover, DPV CHv-BAC- ΔgJ virus replicated 10 times less efficiently than DPV CHv-BAC- gJR and DPV-CHv-BAC. The conclusion is that although gJ gene is unessential to DPV, the deletion of this gene could influence viral replication at late stage of infection.

### Plague morphology of mutant viruses and relative plague area measurements

**Figure 5:**
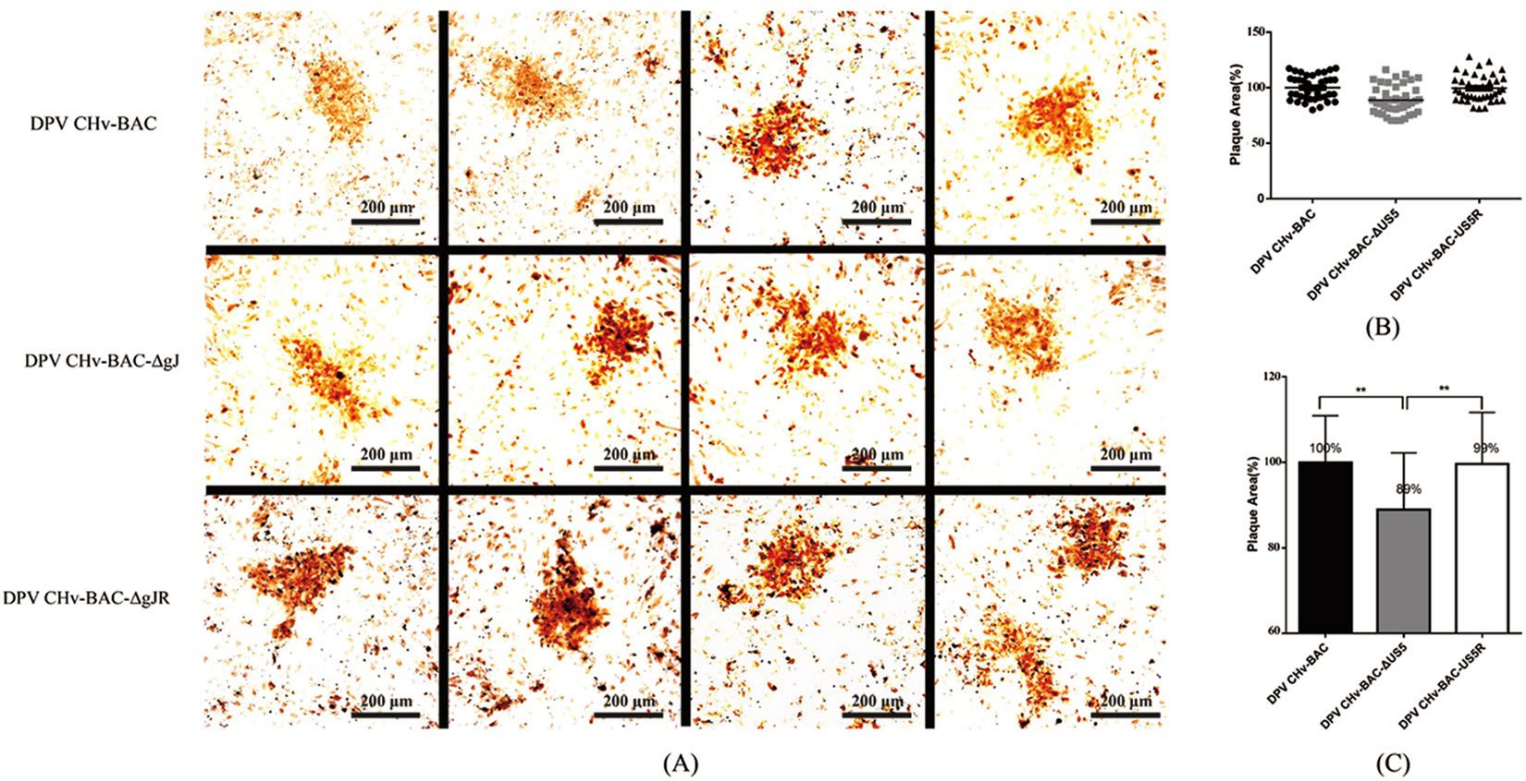
Plaque phenotypes of parental and mutant viruses. (**A**) Confluent DEF cells monolayers were infected with each virus at a 100 TCID_50_, and viral plaques were visualized at 48 hpi by immunohistochemistry as described in Materials and Methods. (**B**) Thirty different viral plaques were randomly selected, imaged, measured, and statistically analyzed as described in Materials and Methods.

Envelop proteins of alpha-herpesvirus play an important role on viral cell-to-cell spreading or plaque forming. We performed plaque morphology assays to explore whether the gJ-deleted viruses had some effects on the transmission of viruses between adjacent cells. The plaque morphologies of the ΔgJ and ΔgJR mutant viruses were examined in DEF cells. As expected, the DPV CHv-BAC parental virus and the DPV CHv-BAC-gJR mutant viruses produced plaques that were similar in size to each other and, on average. The plaques produced by DPV CHv-BAC-ΔgJ mutant viruses were approximately 11% samller than the parental virus plaques. To better assess the virus plaque sizes produced by individual mutant viruses, 30 randomly chosen viral plaques were selected and statistically analyzed as described in Materials and Methods. This analysis confirmed that the gJ-deleted mutant virus slightly reduced plaque size in comparison to that of the parental virus. Meanwhile, these data showed a statistically significant difference in mean plaque areas of the parental and recombinant viruses (t-test, p < 0.05) (Fig. 5).

### Ultrastructural characterization of parental and mutant viruses

The herpesvirus viral life cycle contains the following major steps: entry into the host cell, expression and replication of viral genes, virion assembly, and egress of the new generation of viral particles. The entire process takes approximately 18 to 20 hours in permissive cells^3,21^. Thus, there was a higher opportunity to observe viral ultrastructural phenotypes and life process stages at 36 hpi. Furthermore, according to the growth curve of the recombinant virus, at 36 hpi, its intracellular titer was similar to the parental virus, but its extracellular titer was significantly lower. Meanwhile, the virus extracellular titer gap narrowed at 48 hpi. Moreover, the cells did not show significant rupture and lesions at 36 hpi, but observed at 48 hpi (not shown). In the state of cell fragmentation, it is not conducive to observe the life process of recombinant virus in cells via transmission electron microscopy.

**Figure 6:**
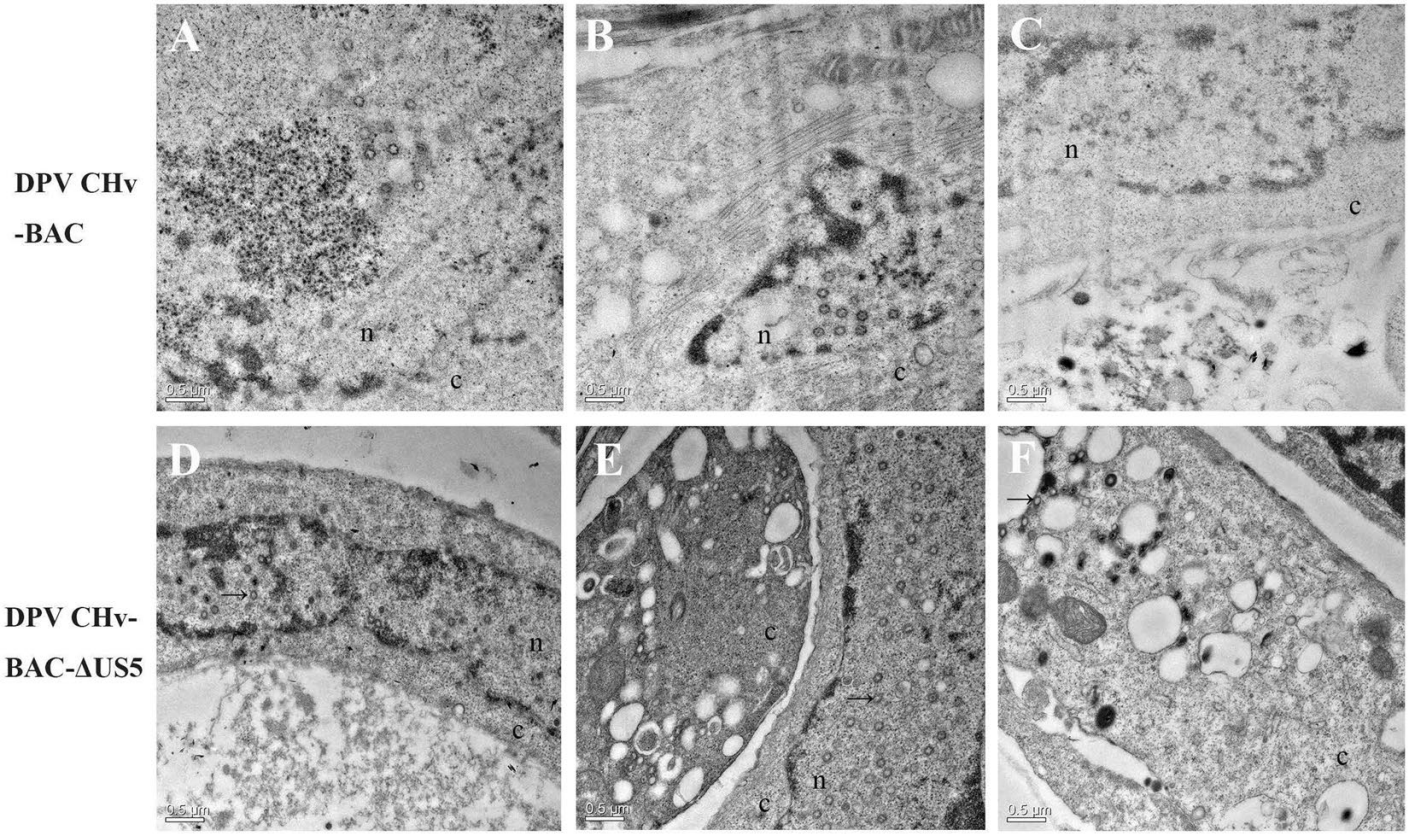
Ultrastructural morphologies of mutant viruses. Electron micrographs of DEF cells infected at an MOI of 2 with different viruses and processed for electron microscopy at 36 hpi are shown. (**A**,**B**,**C**) Showed the DEF cells infected DPV CHv-BAC. (**D**,**E**,**F**) Showed the DEF cells infected DPV CHv-BAC-ΔUS5. Nucleus (n) and cytoplasm (c) are marked.

To test whether the deletion of DPV gJ caused a defect in viral assembly and maturation. The ultrastructural phenotypes of recombination viruses relative to the parental virus were investigated at 36 hpi utilizing transmission electron microscopy, visually examining more than 30 individual cells. As expected, the parental virus did not exhibit any apparent defects in nuclear virion assembly or cytoplasmic virion envelopment, as evidenced by the presence of fully enveloped virions intracellularly. Although the empty capsids could be observed on DPV CHv-BAC infected cells, a large amount of nucleic acids could also be found next to these particles (Fig. 6A,B and C). On the contrary, ultrastructural visualization of DEF cells infected with the gJ-deleted mutant viruses revealed nuclear and cytoplasmic defects in virion assembly and envelopment. The most-pronounced effects, produced by the ΔgJ mutant virus, were that numerous unenveloped and enveloped empty capsids were found in nuclear and cytoplasm of infected cells, which may cause the formation of immature of non-infectious virions (Fig. 6D,E and F).

**Figure 7:**
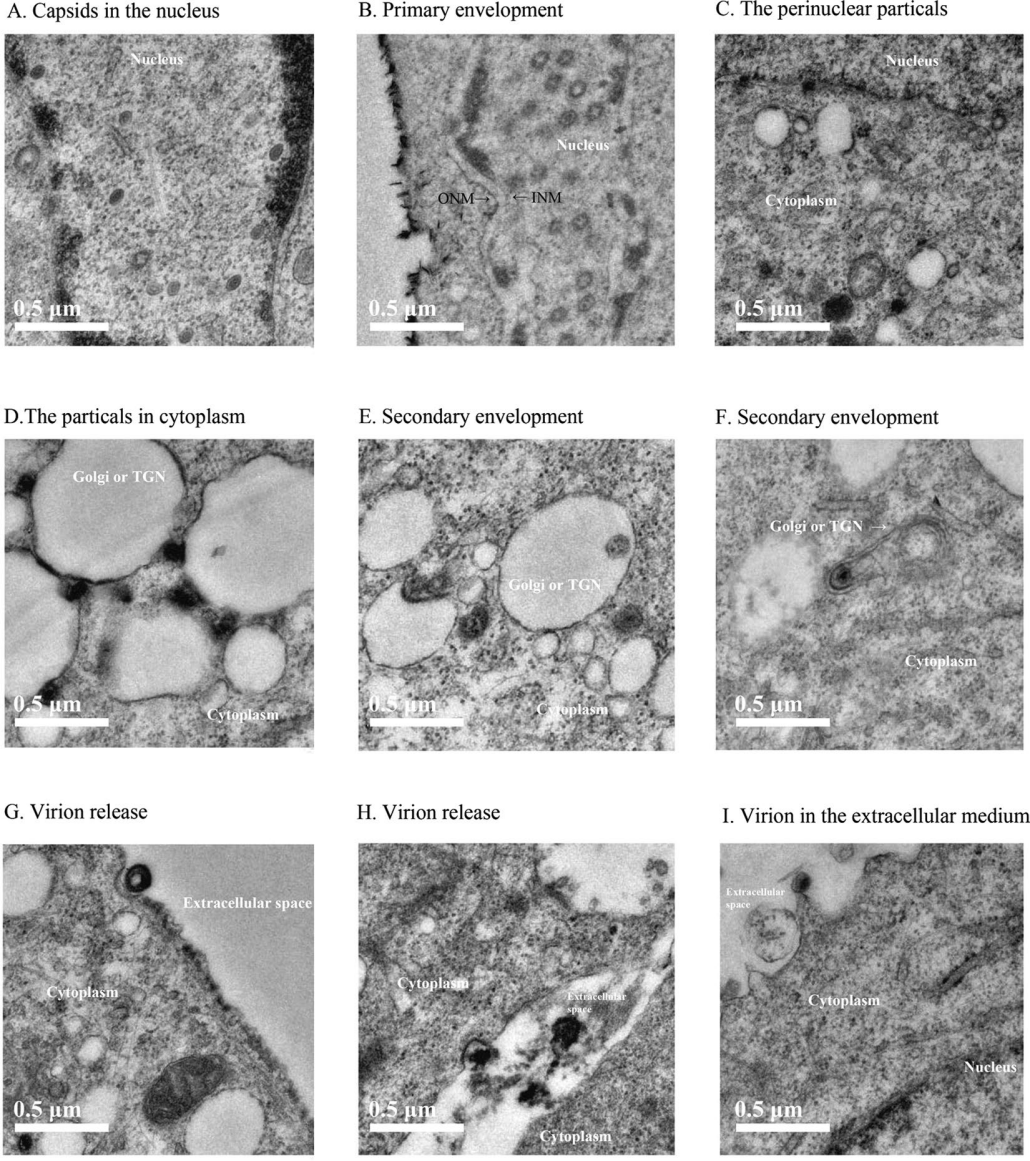
Electron micrographs of the steps of gJ-deleted mutant virus lifecycle. (**A**) Capsids in the nucleus. (**B**) Primary envelopment, showing the close apposition of the particles and the inner nuclear membrane (INM). (**C**) Primarily enveloped particles present within the perinuclear space. (**D**) Particles in the cytoplasm, showing the close apposition of the particles and the Golgi or trans-Golgi network (TGN). (**E**) Initial steps of secondary envelopment. The unenveloped capsids in the cytoplasm interacted with TGN membrane and was being wrapped in these membranes. (**F**) Final steps of secondary envelopment. Enveloped particles were present with the TGN-derived membranes. (**G**) and (**H**) Virions release. Enveloped virions are transported to cell surface and released. (**I**) Virion in the extracellular medium.

The alpha-herpesvirus share some strategies for the lytic replication cycle, which mainly includes the viral DNA replication in nucleus, capsid assembly and egress from the nucleus, the envelopment of viral particles in the cytoplasm and the exocytosis of mature virions. During the viral replication cycle, the viral glycoproteins mainly played important roles on viral envelopment, which interact with the viral tegument protein to drive this budding. Ultrastructural visualization of DEF cells infected with the mutant viruses without the expression of gJ revealed that the process of viral replication could work. As the result showed, the gJ-deleted viral nucleocapsids in the nuclear (Fig. 7A) bound onto the surface of inner nuclear membranes (Fig. 7B), and then these perinuclear enveloped particles fused with outer nuclear membranes (Fig. 7C) to enter the cytoplasm, coated-tegument capsids in the cytoplasm bound onto the surfaces of TNG membranes that contained glycoproteins to produce envelopment virions (Fig. 7D,E and F), and the envelopment particles were released from cells (Fig. 7G,H and I).

## Discussion

Alpha-herpesvirus encodes at least 12 glycoproteins which play important roles in the virus lifecycle, including virus-induced cell fusion, virion assembly and viral egress^9^. Over the years, a number of gene-deleted mutant virus based on infectious BAC clone have been constructed and characterized. Previously studies showed that the lack of expression of gB, gH, or gL alone has drastically effect on viral maturation, which is necessary for viral replication, while HSV-1, EHV-1 or other alpha-herpesvirus could replicate and produce infection progeny virus without the expression of other glycoproteins, such as such as gE, gI, gK and so on^22–24^. As a non-conserved gene among the alpha-herpesvirus, the specific characteristic and function of glycoprotein J are just a little known. Previously, studies of HSV-1, ILTV, EHV-1 and EHV-4 gJ suggested that the viruses without expression of gJ could replicate and proliferate^25–27^, but it had different decrease on virus titer except HSV-1^28^.

In this study, based on the construction of infectious BAC clone containing the genome of DPV, experiments were conducted to elucidate the function of the deletion of gene US5, which leads to the absence of gJ from the DPV. The salient findings of these experiments were that the DPV gJ is important for virus growth *in vitro*, and that gJ is slightly involved in direct cell-to-cell spread and in virion maturation. These above conclusions were drawn based on the finding that the gJ-deleted DPV CHv-BAC mutant, DPV CHv-BAC-ΔgJ, replicated to significantly decreased titers in DEF cells, which exhibited a 10-fold reduction of total titers when compared to parental virus and a 4-fold reduction of endocellular titers, and that especially the production of extracellular infectivity was affected. This means that the DPV gJ did not only have some negatively influence on viral replication, but also slightly mediate viral egress. What’s more, the gJ-deleted mutant virus exhibited an approximately 11% reduction in mean plaque diameters when compared to parental or gJ-revertant virus. Furthermore, more immature virions, non-nuclear particles in nuclear and cytoplasm, were observed by electron microscope, and lower cytoplasmic virion envelopment level. Although numerous immature particles were found, the replication cycle of gJ-deleted mutant virus have still worked.

Ultrastructural examination of cells infected with gJ-deleted mutant viruses revealed that the recombination virus exhibited no appreciable defects in cytoplasmic virion envelopment, as also evidenced by the presence of fully enveloped virions in extracellular spaces and secondary-enveloped process in the cytoplasm of infected cells. Surprisingly, the ΔgJ mutant virus exhibited appreciable defect in viral nuclear assembly. Previously studies showed that glycoproteins of herpesvirus mainly played roles in cytoplasmic virion envelopment^10,22^. What’s more, gB, gD and the heterodimer gH-gL, also found in the nuclear members, were confirmed that these glycoproteins could participate in de-envelopment^29,30^. However, there is no evidence that glycoproteins of herpesvirus have effects on viral nuclear assembly^21,31^. This study is the first to show the result that the absence of DPV gJ exhibited a certain degree of effects in viral nuclear assembly, but the mechanism has not been uncovered and still needs to explore. In summary, a marked replication defect was shown after deleting DPV CHv gJ, slightly influencing the efficiency in cell-to-cell spread and virus egress. In combination with analyses of section of infected cells by electron microscopy, the mechanisms and functions of DPV gJ are complex and still need to be exposed.

## Materials and Methods

### Cells and Viruses

The duck embryo fibroblast (DEF) monolayer was incubated at 37 °C with 5% CO_2_ in Minimal Essential Medium (MEM, Gibco, Grand Island, NY) supplemented with 10% newborn calf serum (NBS, Gibco, Grand Island, NY), 100 U/ml penicillin and 100 μl/mg streptomycin. For virus infection, MEM supplemented with 3% NBS was used^32^. DPV CHv strain was separated and preserved in the laboratory.

### Construction of DPV mutant viruses

Mutagenesis was constructed in *E. coli* DH10B by using the Red recombination mutagenesis system with synthetic oligonucleotides^33^ implemented on the bacterial artificial chromosome (BAC) plasmid pBeloBAC11 carrying the DPV CHv genome^34^. The DPV CHv-BAC-ΔgJ virus was constructed by deleting the whole US5 open reading frame (ORF). Moreover, the ΔgJ recombinant virus was used as the backbone for construction of the DPV CHv-BAC-gJR reverse mutant by restoring the US5 ORF. Synthetic oligonucleotides used to mutagenize each targeted gene are shown in Table 1. Specifically, the 5′ end of the forward primer for each mutagenesis contains 56 bp of homologous sequence upstream of the site of mutation, and 20 or 21 bp at the 3′ ends correspond to the kanamycin resistance (KanR) gene (Table 1). A 1590 bp PCR fragment containing the KanR gene flanked on both sides by gJ sequence was amplified from the pKD4 vector by the primers.

**Table 1:** Primers used in this paper.

No.	Primers	Sequence (5′-3′)	Product
1	sopB-for sopB-rev	attcgttaattgcgcgcgtagg gaatattcaggccagttatgct	sopB
2	repA-for repA-rev	catggcggaaacagcggttatc atgtatgagaggcgcattggag	repA
3	ΔgJ/gJR-for ΔgJ/gJR -rev	tttatattgacgcggaatgtt cgctgagtatttattcatttcct	ΔgJ/gJR identification product
4	ΔgJ-Kana-for ΔgJ-Kana-rev	gagtaatttaatgcaagcgatgtaggcctcctgtcgtagtccttatctcatgcagggtgtaggctggagctgcttc aacaacaacagaactgtaatgggtacattaaacatacgcgcatatacatattgccgccatatgaatatcctccttag	Kana gene flanked by homology arms of gJ
5	gJR-gJ-for gJR-gJ -rev	agagtaatttaatgcaagcga gaagcagctccagcctacactcataccatacaaaggcat	gJ fragment with left homology arm of gJ
6	gJR-Kana-for gJR-Kana-rev	atgcctttgtatggtatgagtgtaggctggagctgcttc aacaacaacagaactgtaatgggtacattaaacatacgcgcatatacatattgccgatgggaattagccatggtcc	Kana fragment with right homology arm of gJ

^*^Complementary sequence for overlap PCR.

Maintenance and mutagenesis of the BAC constructs were performed in *E. coli* strain DH10B. Firstly, the recombineering plasmid, pKD46, containing a λ prophage encoding recombination enzymes Exo, Beta, and Gam under a heat-inducible promoter was transformed into E.coli strain DH10B. Secondly, Bacteria carrying the target BAC and pKD46 were grown in 50 ml LB cultures with chloramphenicol (25 μl/ml), ampicillin (100 μl/ml), and L-arabinose (100 μl/ml, Sigma) at 30 °C to an OD600 of 0.5~0.6. Electrocompetent bacteria were transformed with 800 ng of the PRC products. Chloramphenicol-resistant (CmR)/KanR transformants were confirmed sufficient resistance, and then screened by PCR using the identification of primer (Table 1). Positive colonies were grown overnight at 42 °C to cure pKD46. To remove the KanR gene, a single CmR/KanR and ampicillin-sensitive colony was transformed with pCP20 and grown overnight at 30 °C on Cm/Amp plates. Some colonies were selected again in Cm plates at 42 °C and then tested for kanamycin sensitivity and loss of ampicillin resistance^35^. The primarily confirmed colonies were ulteriorly screened by PCR using the identification of primer, and then the PCR products were sequenced to confirm the desired targeting.

### Confirmation of the targeted mutations and recovery of infectious virus

DPV CHv-BAC DNAs (plasmids pΔgJ and pgJR) were purified from 100 ml of BAC cultures with a Qiagen Plasmid Midi Kit (Qiagen, Valencia CA). The plasmids pΔgJ and pgJR were confirmed by PCR Using the identification of primers designed to lie outside of the target mutation site.

Viruses were recovered from cells transfected with BACs as follows: DEF cells were grown to 70–90% confluent in 6-well or 12-well plates. Cells were transfected with BAC DNAs mixed with Lipofectamine 3000 in Opti-MEM medium recommended by the manufacturer (Invitrogen). After 6 h of incubation at 37 °C, the medium was removed from the transfected cells, and the cells were washed with phosphate-buffered saline (PBS) and then fresh MEM with 3% NBS was added. After incubating cells for 2–4 days at 37 °C, virus stocks were collected and designated as passage 0 (P_0_). To remove the transient expression of EGFP, the virus of P_0_ was blindly passaged. Passage P_3_ viruses were used for all experiment described in the manuscript.

### One-step viral growth kinetics

One-step growth curves were performed as follows. Sub-confluent (85 to 90%) DEF cell monolayers grown in 24-well cell culture dish were infected with each virus at 37 °C at 1000 50% Tissue Culture Infection Does (TCID_50_). After adsorption at 37 °C for 2 h, the cell monolayers were rinsed with PBS overlaid with 2 ml MEM supplemented with 3% NBS (set as 0 h on the time scale), and then returned to 37 °C humidified incubator (5% CO_2_). At selected time points (12, 24, 36, 48, 72, and 96 h post-infection [h.p.i]), supernatants and cell pellets were separated at different times post-infection and stored at −20 °C. The samples were frozen and thawed three times, and virus titers were determined by TCID_50_ on DEF cells. All experiments repeated three times.

### Plague morphology of mutant viruses and relative plague area measurements

Near-confluent DEF cell monolayers in 12-well plates were infected with the virus at 100 TCID_50_. After 2 h at 37 °C, medium was discarded and cells were washed with PBS twice. And then 0.5% methylcellulose was added to cover the cells. After 48 h post-infection, cells were washed three times with PBS to remove methyl cellulose medium and fixed with ice-cold 4% paraformaldehyde for 30 min. Photographs of viral plagues were taken at ×200 magnification on microscope. Thirty randomly selected plagues were imaged in this manner for each of the parental and recombination viruses under consideration.

### Electron Microscopy

The ultrastructural morphology of virions within infected cells was examined by transmission electron microscopy essentially as described previously^36,37^. Cell monolayers were infected with the indicated virus at a multiplicity of infection (MOI) of 2. All samples were prepared for transmission electron microscopy examination according to previously reports^38^. Briefly, DEF cells were washed with PBS at 36 hpi and fixed with 2.5% glutaraldehyde at 4 °C for 2 h. After that, the fixed adherent cells were collected by scraping from the plates and then centrifuged at 10,000 rpm/min for 1 h. Then the pellets were mixed with 2% low melting-temperature agarose at 37 °C, and centrifuged at 6000 rpm/min for 10 min. Samples were post-fixed in 1.0% osmium tetroxide. After a stepwise dehydration in acetone, samples were embedded in epoxy resin 618 and polymerized at 80 °C for 72 h. Then, 50 nm ultra-thin sections were prepared, collected on grids and stained with uranyl acetate and lead citrate for subsequent examination with the Tecnai G^2^ F20 transmission electron microscope.
