# Author Correction: Duck plague virus Glycoprotein J is functional but slightly impaired in viral replication and cell-to-cell spread

**DOI:** 10.1038/s41598-018-24845-7

**Published:** 2018-04-19

**Authors:** Yu You, Tian Liu, Mingshu Wang, Anchun Cheng, Renyong Jia, Qiao Yang, Ying Wu, Dekang Zhu, Shun Chen, Mafeng Liu, XinXin Zhao, Shaqiu Zhang, Yunya Liu, Yanling Yu, Ling Zhang

**Affiliations:** 1 Institute of Preventive Veterinary Medicine, Sichuan Agricultural University, Wenjiang, Chengdu, Sichuan 611130 P.R. China; 2 Key Laboratory of Animal Disease and Human Health of Sichuan Province, Sichuan Agricultural University, Wenjiang, Chengdu, Sichuan 611130 P.R. China; 3 Avian Disease Research Center, College of Veterinary Medicine, Sichuan Agricultural University, Wenjiang, Chengdu, Sichuan 611130 P.R. China

Correction to: *Scientific Reports* 10.1038/s41598-018-22447-x, published online 06 March 2018

In Figure 3B, the upper and the lower immunofluorescence images are the same. The correct Figure 3 appears below as Figure 1.

**Figure 1:**
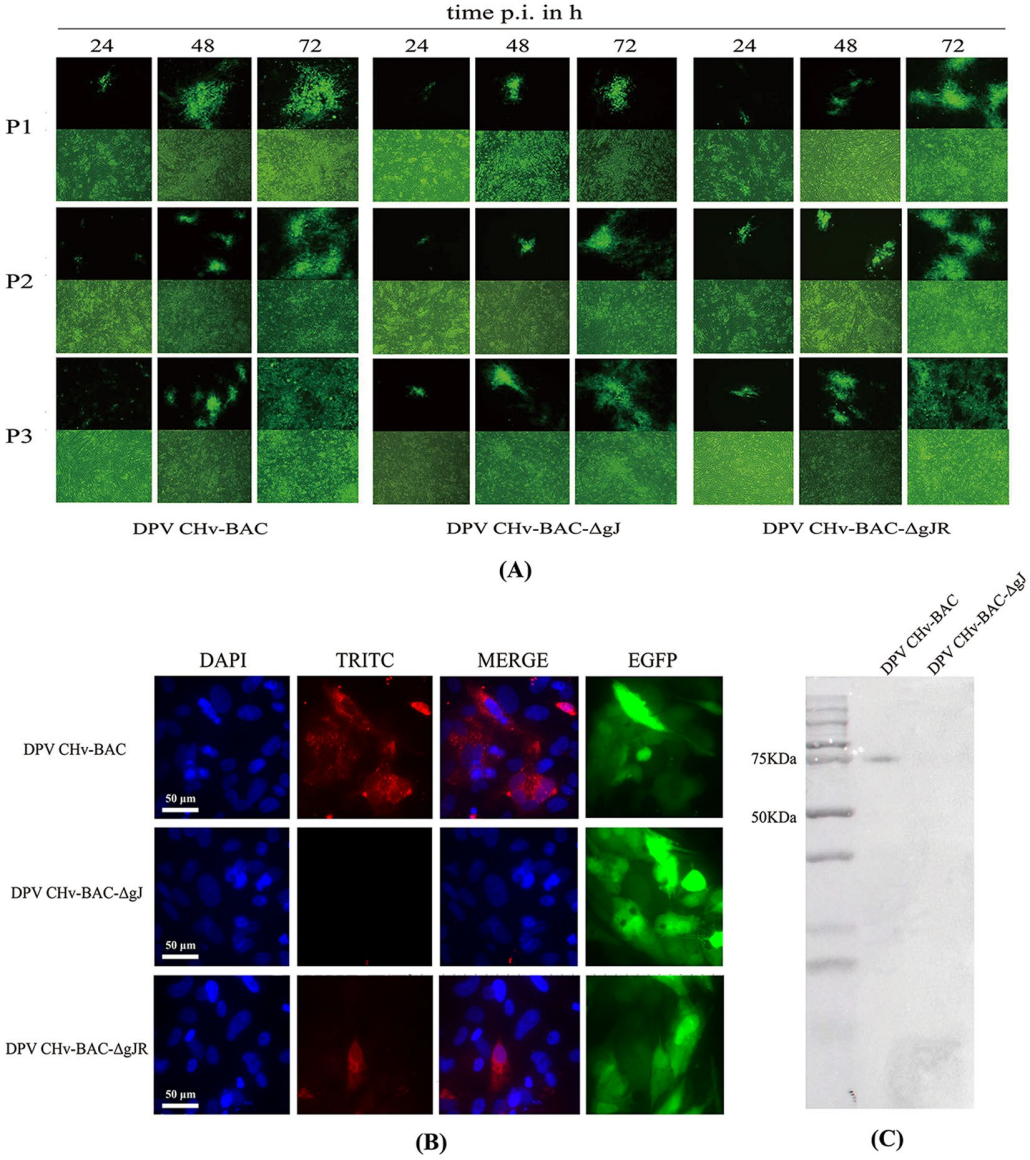
Rescue mutant viruses and Identification of gJ expression. (**A**) Purification and enrichment of mutant viruses. Purification and enrichment of mutant viruses were obtained by the three times passage after transfection. (**B**) Immunofluorescence detection of gJ expression. DEF cells were infected at 1000 TCID50, and gJ expression was detected by indirect immunofluorescence at 36 hpi. Rabbit anti-gJ were used as primary antibody, and goat anti-rabbit IgG TRITC were used as secondary antibody. (**C**) Anti-gJ monoclonal antibody (MAb) was used to detect gJ via western immunoblot analysis. DPV CHv-BAC-ΔgJ infected DEF cells were detected as parental virus.

